# Association between health indifference and problem drinking using a nationwide internet survey

**DOI:** 10.1265/ehpm.22-00306

**Published:** 2023-04-18

**Authors:** Mami Wakabayashi, Hirono Ishikawa, Yoshiharu Fukuda, Hiroyasu Iso, Takahiro Tabuchi

**Affiliations:** 1 Institute for Global Health Policy Research, Bureau of International Health Cooperation, National Center for Global Health and Medicine, Tokyo, 162-8655, Japan; 2 Teikyo University Graduate School of Public Health, 2-11-1, Kaga, Itabashi-ku, Tokyo, 173-8605, Japan; 3 Cancer Control Center, Osaka International Cancer Institute, 541-8567, Osaka, Japan; 4 The Tokyo Foundation for Policy Research, 106-6234, Tokyo, Japan

**Keywords:** Alcohol Use Disorder Identification Test, Hazardous alcohol use, Health consciousness, Health inequality, Japan

## Abstract

**Background:**

Little is known about the vulnerable populations and problem drinking in terms of health inequality. This study aimed to investigate the relationship between health indifference estimated by Health Interest Scale (HIS) and problem drinking identified by the Alcohol Use Disorder Identification Test (AUDIT).

**Methods:**

A cross-sectional study was conducted utilizing data from a nationwide internet survey in Japan in 2022. The number of total participants was 29,377, with 49% of them being male, and the mean age was 47.9 (±17.9) years. The participants were categorized into the following groups based on the quintiles of HIS score: health indifference (0–16), low health interest (17–20), middle health interest (21–22), middle-high interest (23–26) and high health interest (27–36) groups. Problem drinking was identified as AUDIT score of ≥8 points.

**Results:**

The association between health indifference and problem drinking was explored through logistic regression with adjustment for various socioeconomic status, such as education, income level, and occupation; the adjusted odds ratio (aOR) was 1.72 [95% confidence interval (CI): 1.51–1.95].

**Conclusion:**

Health indifferent or lower health interest groups were a vulnerable population for problem drinking, regardless of their socioeconomic status. It could be useful to identify the health indifferent group through HIS and to monitor the impact of health intervention for this group for the reduction of health inequality.

**Supplementary information:**

The online version contains supplementary material available at https://doi.org/10.1265/ehpm.22-00306.

## Graphical Abstract

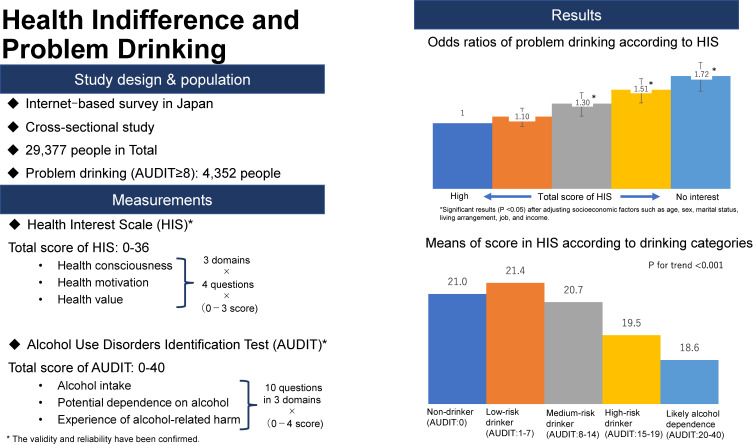

## 1 Background

Harmful alcohol use is unevenly distributed across society, such as depending on gender, socioeconomic status, and ethnicity [1, 2]. Harmful use of alcohol is accountable for 6.8% and 2.2% of total age-standardized deaths for males and females, respectively [3]. Harmful alcohol use is one of the major risk behaviors associated with diseases such as cancer, coronary heart disease, and cardiovascular disease, which often leads to death [3–6]. Although various health promotion strategies to prevent harmful alcohol use are conducted, little research has examined the effectiveness of interventions from health inequality [7]. The theory described that contemporary behavioral–health promotion strategies tend to generate significantly less or little improvement in individuals with low socioeconomic status or other disadvantaged groups [8, 9]. As a result of health intervention, individuals may entrench or exacerbate inequality in health behavior and health outcomes, such as smoking cessation, pediatric obesity, and salt intake intervention [10–12]. Additionally, the association between socioeconomic status and harmful alcohol use has bidirectional influences [2]. For instance, hazardous alcohol users may face loss of jobs, family disruption, interpersonal violence, mental health issues, stigmatization, and barriers to accessing health care.

Socioeconomically disadvantaged people are generally associated with risky health behavior such as heavy drinking [13]. However, socioeconomic characteristics of harmful alcohol use in Japan were identified in men with a high household income, were married, and were managers or professionals, which generally have a high socioeconomic status [14, 15]. The other study showed that the motivation for drinking alcohol among middle-aged men indicated the social norm that alcohol drinking is important as a communication tool in business in Japan [16]. The age-standardized percentage of problem drinking defined by the Alcohol Use Disorders Identification Test (AUDIT) was 21.4% for men and 4.5% for women, according to results from the Periodical Nationwide Surveys (2018) in Japan [17]. A vulnerable population for problem drinking in Japan may have been influenced by factors other than low socioeconomic status.

Most theoretical models of health intervention based on health behavior change begin with the notion that an individual is motivated to prevent disease or improve health [18]. The first process of change in the Transtheoretical Model of health behavior change, which was proposed by Prochaska in 1984, is “consciousness raising” [19]. In other words, individuals who are indifferent to health are at a state before health consciousness raising and they are not ready to change their health behavior [20]. That health indifferent group may be vulnerable for health intervention based on health behaviors changing models. Therefore, this study aimed to identify the characteristics of the health indifferent group.

The Japanese government set up the “National Plan for Extension of Healthy Life Expectancy” as a national health strategy in 2019 with a goal of ≥3 years of extension of healthy life expectancy by 2040 [21]. To achieve the goal of this national plan, the government encourages health promotion programs like nudging that could be targeted at the health-indifferent group. Nudging is increasingly used in public health interventions in western societies to enhance health-promoting behaviors [22]. However, the concept of the health indifferent group and the method of identifying the health indifferent group are not defined.

A Japanese research group developed the Health Interest Scale (HIS), and its validity and reliability have been confirmed [23]. In the previous study, the mean score of HIS did not differ between nondrinkers and drinkers. Based on this scale score, we defined the health indifference and investigated problem drinking at different levels of HIS. We hypothesized that the health indifference were associated with problem drinking. Our study aimed to confirm the external validity of HIS as a screening tool for identifying a health-indifferent group at high risk of problem drinking using a large sample size.

## 2 Methods

### 2.1 Data source and study population

#### 2.1.1 Internet survey

A cross-sectional study was conducted by utilizing data from the Japan Society and New Tobacco Internet Survey (JASTIS)—a large internet-based cohort study that focuses on tobacco issue since 2015 and has expanded to various health behaviors since 2021. The details of the JASTIS have been previously published [24]. The survey for this study was conducted in February 1–28, 2022 in Japan. This web-based, self-reported questionnaire survey was administrated by a large internet research agency, Rakuten Insight, Inc., which pooled approximately 2.2 million panelists as of September 2022 [25]. The survey requests were sent by the research agency to the panelists, who were each selected by sex, age, and prefecture. The panelists who consented to participate accessed the designated website and responded to the survey. The participants were given the option to not respond to any part of the survey or discontinue it altogether at any point. The survey was closed when the target number of respondents for each sex, age, and prefecture was met. The survey included 28,776 out of 39,998 participants who were eligible as cohort study participants (participation rate was 71.9%). Lastly, 4,224 participants were included as new cohort members, resulting in a total of 33,000 participants in JASTIS 2022.

#### 2.1.2 Managing data quality and generating the study population

**Fig. 1:**
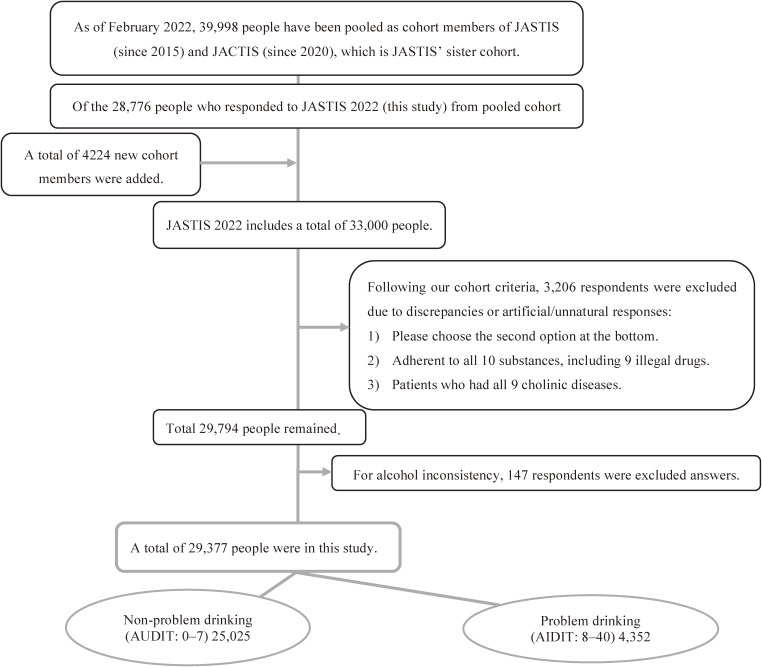
The flow diagram of the study population The figure shows the steps used in the selection of the members of this study. Information on the two cohort studies is available on the website: JUSTIS (https://jastis-study.jp/), and JACSIS (https://jacsis-study.jp/index.html).

To ensure data quality, respondents with discrepancies or artificial/unnatural responses were excluded from the study [24]. In this regard, the following three items were used to detect discrepancies: (1) “Please choose the second from the bottom”; (2) choosing “yes” in all the questions for using alcohol and nine drugs, including illegal drugs; and (3) choosing “yes” in all the questions for having nine chronic diseases. Moreover, 753 respondents who had inconsistent answers to alcohol-related questions were excluded. As shown in Fig. 1, the total number of participants was 29,377, and 19,677 current drinkers were identified which allowed us to test on the hypothesis.

### 2.2 Study design and measures

We explored the association between health indifference and problem drinking for the main analysis.

#### 2.2.1 Explanatory variables for HIS categories

The HIS comprised of 12 items with three factors, such as health consciousness, health motivation, and health values [23]. Each item in the HIS had a score of 0–3 points, and the total scores ranged 0–36 points. The details of the questionnaire are displayed in Additional File 1. We divided the total population into quantile and defined health indifferent group as having a score within the lowest quintile [26]. As a result, the HIS scores were categorized as explanatory variables, which are as follows: “health indifference (no interest) (0–16),” “low health interest (17–20),” “middle health interest (21–22),” “middle-high health interest (23–26),” and “high health interests (27–36).”

#### 2.2.2 Outcome variables for drinking categories

Problem drinking was identified by the Alcohol Use Disorders Identification Test (AUDIT), which is developed by the World Health Organization and is the most effective screening tool to identify individuals with alcohol-related problems [27]. The AUDIT is a 10-item screening measure that assesses alcohol use during the previous 12 months [28, 29]. The AUDIT, translated into Japanese, was validated to identify hazardous alcohol use and alcohol use disorders as a screening tool [30]. Each item in the AUDIT has a score of 0–4 points, except items 9 and 10, which investigated about alcohol-related injury or violence with scores of 0, 2, or 4 points. The total scores range from 0 to 40 points. The details of each question are described in Additional File 3.

The participants were categorized into two groups based on the total score: nonproblem drinking (score: 0–7) and problem drinking (≥8) [30]. Problem drinking (≥8) was defined as the target population that needed advice for the reduction of hazardous drinking in Japan [17, 28].

#### 2.2.3 Demographics and potential health factors related to alcohol use

The demographic data obtained were as follows: age, sex, educational level, marital status, current living arrangements, job, and equivalent annual household income. Educational level was categorized as low (graduated from high school or lower), middle (graduated from vocational or junior college), and high (graduated from university or higher). Marital status was categorized as married, single, and divorced/widowed. The current living arrangements reported about whether the participant lived with someone or alone. The job was categorized as executive/manager, regular employee, self-employed, nonregular employee, no main job as the individual is a student, no main job as the individual is a retiree, only housework, and unemployed. Equivalent annual household income is the household income divided by the square root of the number of household members. This factor is categorized as <2 million yen, 2–4 million yen, 4–6 million yen, 6–10 million yen, ≥10 million yen, and do not know/do not want to answer.

### 2.3 Statistical analyses

Statistical analyses were performed using Stata MP version 15 (StataCorp LLC, College Station, Texas, USA). Continuous variables were presented as the means and standard deviations (SD), whereas categorical variables were presented as proportions. The test of a linear trend was conducted for the score of HIS and drinking categories. We determined the variations in the means and proportions of the demographic data and the potential health factors according to the AUDIT scores for each category. Then, multivariable logistic regression was performed to examine the relationship between health indifference and problem drinking. Model 1 was univariable, whereas Model 2 was adjusted for the sociodemographic factors shown in Table 1. Finally, the adjusted odds ratios (aORs) and 95% confidence intervals (CI) for problem drinking were reported. All statistical tests conducted were two-sided, and p-value < 0.05 was considered statistically significant.

**Table 1:** The characteristics of participants according to sociodemographic factors

	**Total**	**The drinking categories**	

		**Identifying Problem drinking** **N = 29,377**	

		**Non-problem** **(AUDIT:0–7)**	**Problem** **(AUDIT:8–40)**

	**N = 29,377**	**N = 25,025**	**N = 4,352**

	**N (%)**	**N (%)**	**N (%)**
Age, year (Mean, SD)	47.9(17.9)	47.9(18.1)	48.1(16.4)
39 or less years	10457(36)	9039(36)	1418(33)
40–59 years	9773(33)	8095(32)	1678(39)
60 or over years	9147(31)	7891(32)	1256(29)
Sex			
Men	14389(49)	11165(45)	3224(74)
Women	14988(51)	13860(55)	1128(26)
Education			
Low	8858(30)	7574(30)	1284(30)
Middle	5977(20)	5352(21)	625(14)
High	14542(50)	12099(48)	2443(56)
Marital status			
Marriage	16829(57)	14138(57)	2691(62)
No marriage	9875(34)	8569(34)	1306(30)
Divorced/Widowed	2673(9)	2318(9)	355(8)
Living alone	6557(22)	5459(22)	1098(25)
Job			
Executive/management	2973(10)	2094(8)	879(20)
Permanent employee	8688(30)	7254(29)	1434(33)
Self-employee	1658(6)	1314(5)	344(8)
No-regular employee	5096(17)	4466(18)	630(14)
Students	2057(7)	1851(7)	206(5)
Retirement	919(3)	763(3)	156(4)
Houseworker	4474(15)	4267(17)	207(5)
Unemployed	3512(12)	3016(12)	496(11)
Income			
Under 2 million yen	4601(16)	3953(16)	648(15)
2–4 million yen	10239(35)	8714(35)	1525(35)
4–6 million yen	4856(17)	4029(16)	827(19)
6–10 million yen	2974(10)	2363(9)	611(14)
≥10 million yen	628(2)	458(2)	170(4)
Don’t know/Don’t want to answer	6079(21)	5508(22)	571(13)

### 2.4 Ethical approval

All the procedures were conducted in accordance with the ethical standards of the Helsinki Declaration of 1975 (revised in 2013). The Research Ethics Committee of the Osaka International Cancer Institute has reviewed and approved the present study’s protocol on January 8, 2020 (approval No. 1611079163-2). All the participants provided their informed consent before responding to the online questionnaire. Furthermore, the internet survey agency respected the Act on Protection of Personal Information in Japan. As an incentive, credit points (known as “E-points”), which can be used for internet shopping and cash conversion, were provided to the participants.

## 3 Results

### 3.1 Participants’ characteristics

Of the 29,377 participants, 49% (n = 14,389) were men, the mean age was 47.9 (±17.9) years, and age range was 15–81 years (Table 1). The number of people with problem drinking (AUDIT score of ≥8) was 4,352 (15% of the total participants). Additionally, their mean age was 48.1 (±16.4) years. The age-standardized percentage of problem drinking was 21.8% for men and 6.6% for women. The sociodemographic characteristics with the high proportion of responses in problem drinking as compared to nonproblem drinking were age of 40–59 years, male, higher educational level, married, living alone, executive/manager, permanent employee, self-employee, income level of 6–10 million yen, and income level of ≥10 million yen. Additional File 2 described the characteristics of HIS categories. The mean HIS score was 21.1 (±5.8). The health indifferent group was younger ages of ≤39 years, male, a low educational level, unmarried, and living alone, which were inversely associated with health interest.

Table 2 shows the proportion of problem drinking according to quintiles of HIS. The proportion of health indifferent group was higher in problem drinkers than in nonproblem drinkers (26% vs 20%). The means and standard deviations of HIS score were 20.1 (±5.8) for problem drinking. Means and standard deviations of HIS score according to drinking categories are shown in Additional File 4. The mean of HIS score were high in non-drinkers and low-risk drinkers, and relatively lower in medium-risk drinkers, high-risk drinkers, and likely alcohol dependent (p for trend < 0.001). Likewise, the HIS components of health consciousness, health motivation, and health value showed similar trend.

**Table 2:** The distribution between Health Interests Scale (HIS) groups and drinking categories

	**Total**	**Drinking categories through AUDIT**	

		**Non-problem** **(AUDIT:0–7)**	**Problem** **(AUDIT ≥ 8)**

	**N = 29,377**	**N = 25,025**	**N = 4,352**
The mean score of HIS (SD)	21.1(5.8)	21.3(5.8)	20.1(5.8)

HIS groups	N (%)	N (%)	N (%)

Health indifference (HIS:0–16)	6195(21)	5043(20)	1152(26)
Low health interest (HIS:17–20)	7362(25)	6151(25)	1211(28)
Middle health interest (HIS:21–22)	4070(14)	3498(14)	572(13)
Middle-high health interest (HIS:23–26)	6689(23)	5857(23)	832(19)
High health interests (HIS:27–36)	5061(17)	4476(18)	585(13)

### 3.2 The association between health indifference and problem drinking

Table 3 presents odds ratios of problem drinking according to quintiles of HIS. Health indifference or lower health interest was associated with problem drinking as compared with high health interest group after adjusting for potential confounding factors. The adjusted odds ratio (aOR) between health indifference and problem drinking was 1.72 (95% confidence interval [CI]: 1.51–1.95). Being a man and married, having low education, living alone, being an executive/manager, and having high income were all associated with problem drinking.

**Table 3:** Odds ratios of problem drinking according to health interest scale (HIS)

	**Problem drinking** **(8 or over by AUDIT) among total participants (N = 29,377)**	

	**Model 1**	**Model 2**

	**OR (95% CI)**	**aOR (95% CI)**
Health interest score by HIS		
Health indifference (0–16)	**1.75(1.57–1.95)**	**1.72(1.51–1.95)**
Low health interest (17–20)	**1.51(1.35–1.67)**	**1.51(1.34–1.71)**
Middle health interest (21–22)	**1.25(1.10–1.42)**	**1.30(1.13–1.49)**
Middle-high health interest (23–26)	1.09(0.97–1.21)	1.10(0.97–1.25)
High health interest (27–36)	Ref	Ref
Age, year (Mean, SD)		
≤39 years	Ref	Ref
40–59 years	**1.32(1.22–1.42)**	1.07(0.97–1.17)
60 ≥ years	1.01(0.93–1.10)	1.09(0.96–1.24)
Sex		
Women	Ref	Ref
Men	**3.55(3.30–3.81)**	**2.5(2.24–2.70)**
Education		
Low	Ref	Ref
Middle	0.69(0.62–0.76)	**0.81(0.72–0.92)**
High	**1.19(1.10–1.28)**	**0.89(0.82–0.98)**
Marital status		
Married	Ref	Ref
Single	**0.80(0.75–0.86)**	**0.73(0.65–0.82)**
Divorced/Widowed	**0.80(0.71–0.91)**	**0.80(0.68–0.93)**
Living arrangement		
Living with someone	Ref	Ref
Living alone	**1.20(1.12–1.30)**	**1.30(1.17–1.45)**
Job		
Executive/Manager	Ref	Ref
Regular employee	**0.47(0.43–0.52)**	**0.60(0.54–0.67)**
Self-employed	**0.62(0.54–0.71)**	**0.72(0.62–0.86)**
Non-regular employee	**0.34(0.30–0.38)**	**0.61(0.53–0.70)**
Students	**0.27(0.22–0.31)**	**0.56(0.46–0.70)**
Retirement	**0.49(0.40–0.59)**	**0.60(0.48–0.75)**
Housework	**0.12(0.10–0.14)**	**0.27(0.22–0.33)**
Unemployed	**0.39(0.35–0.44)**	**0.57(0.49–0.67)**
Income(equivalent)		
Under 2 million yen	Ref	Ref
2–4 million yen	1.07(0.97–1.18)	0.98(0.88–1.09)
4–6 million yen	**1.25(1.12–1.40)**	0.99(0.88–1.13)
6–10 million yen	**1.57(1.40–1.78)**	**1.21(1.05–1.39)**
>10 million yen	**2.26(1.86–2.75)**	**1.63(1.32–2.02)**

Bold items were significant (p < 0.05).

Model 1: univariable for logistic regression;

Model 2: adjusted factors shown in Table 1 for multivariable logistic regression.

## 4 Discussion

The health indifferent group had several vulnerable factors for health [8], such as younger age, low level of education, unmarried, living alone, and low-income level in our study. The health indifferent group had a higher risk of problem drinking, regardless of their socioeconomic factors. The percentage of problem drinking was similar to that reported in the Periodical Nationwide Surveys (2018).

The recognition of risky drinking is very complex in the society, even though heavy alcohol drinking cause or contribute to the development of many non-communicable diseases. People believe light to moderate drinking is good for health. However, only half of people recognized the level of moderate drinking according to the national evaluation for national health plan which called “Health Japan 21 (the first term)” in 2011 [31]. There is no safety level of alcohol consumption in cancer [32], whereas light to moderate alcohol intake showed a lower risk of total mortality and cerebrovascular diseases [33]. Therefore, it is a limit for individual to build their goal for moderate drinking.

The effectiveness of informing the healthy drinking guideline is limited. For example, young people in Australia consumed alcohol at harmful levels, although they understood of alcohol-related risks perfectly [34]. About 80% of women in Australia consumed alcohol during pregnancy in spite of a guideline for pregnant women [35]. The guideline may potentially have a greater impact on those who are more health literate and who have a higher capacity to implement behavior change [36, 37]. Alternatively, the health indifferent group would not be interested in those healthy guidelines even though a health specialist has informed them of the guidelines as a brief intervention. Widely accepted current health promotion intervention, such as the Transtheoretical Model, were based on self-motivating behavior change [19]. However, environmental approach may have some possibility of changing their health behavior regardless of health interest. For instance, reducing demand via taxation, labeling, or financial incentive and nudge may be effective to reduce engaging in risky health behaviors, such as excessive alcohol drinking [38, 39]. This environmental approach as a health intervention could be meaningful for drinkers to prevent problem drinking regardless of their health interest and it is necessary to evaluate the impact of those interventions among the health indifferent group.

### Strengths and limitations

The main strengths of this study are that it involves a large sample size. Our study found the association between HIS categories and problem drinking, and confirmed the external validity of HIS as a screening tool for identifying a health indifferent group with a high risk of problem drinking. Lastly, both self-reported HIS and drinking pattern based on AUDIT are reliable and valid in Japanese individuals [23, 40].

This study has several limitations. First, due to cross-sectional design of this study, we could not determine the causal direction whether people with health indifference developed problem drinking. A cohort study is necessary to examine the association between health indifference and the risk of problem drinking. Second, our participants may have selection bias. The generalizability of the results was not assured because of the online voluntary survey. The participants in our study had a higher level of socioeconomic status than those included in national census 2020 among those aged 20–79 years; e.g., the proportions of participants with university and higher educational level were 49% and 37%, respectively [41]. Nevertheless, the association between health indifference and problem drinking after adjusting socioeconomic factors as main finding would be generalized. Third, because our survey was conducted in the middle of six-wave outbreaks of COVID-19, we did not examine the change in health interest among the participants before, during, and after the COVID-19 pandemic.

## 5 Conclusion

Health indifferent and low health interest groups had a lower socioeconomic status, which would imply vulnerable populations for health intervention based on the theory of self-motivating behavior change. The health indifferent groups are more likely to have problem drinking, while the higher health interest groups are more likely to control their drinking. The environmental approach as a health intervention could be useful for drinkers to prevent problem drinking regardless of their health interest, and it is necessary to evaluate the impact of such an intervention among the health indifferent group.

## Abbreviations

AUDIT: Alcohol Use Disorders Identification Test
aOR: Adjusted odds ratios
CI: Confidence interval
COVID-19: Coronavirus disease caused by the SARS-CoV-2 virus
HIS: Health Interest Scale
JASTIS: Japan Society and New Tobacco Internet Survey
SD: Standard deviation

## Supplementary information

**Additional File 1.** The Health Interest Scale (HIS) questions and each score of answers. **Additional File 2.** The characteristic of Health Interest Scale (HIS) categories. **Additional File 3.** The Alcohol Use Disorders Identification Test (AUDIT) questions and each score of answers. **Additional File 4.** Means and standard deviations of Health Interest Scale (HIS) according to drinking categories.

## Declarations

### Availability of data and material

The data supporting the findings of this study are available on reasonable request. However, restrictions apply to the availability of these data to protect personal identification and such data are not shared. If any person wishes to verify our data, they may contact the corresponding author.
