# Supplementary material for: Association between health indifference and problem drinking using a nationwide internet survey
Source: Environ Health Prev Med. 2023 Apr 18;28:24. doi: 10.1265/ehpm.22-00306 (PMC10149320; doi:10.1265/ehpm.22-00306)
Supplement: Supplementary file 1 — Additional File 1. The Health Interest Scale (HIS) questions and each score of answers. Additional File 2. The characteristic of Health Interest Scale (HIS) categories. Additional File 3. The Alcohol Use Disorders Identification Test (AUDIT) questions and each score of answers. Additional File 4. Means and standard deviations of Health Interest Scale (HIS) according to drinking categories. [file ehpm-28-024-s001.docx]

Additional File 1. The Health Interest Scale (HIS) questions* and each score of answers

|  | 3 | 2 | 1 | 0 |
| --- | --- | --- | --- | --- |
| Health Consciousness | | | | |
| 1. I’m very self-conscious about my health. | Agree | Somewhat agree | Somewhat disagree | Disagree |
| 1. I’m interested in information about my health. | Agree | Somewhat agree | Somewhat disagree | Disagree |
| 1. I pay attention to changes in my health condition. | Agree | Somewhat agree | Somewhat disagree | Disagree |
| 1. I am more health conscious than people around me. | Agree | Somewhat agree | Somewhat disagree | Disagree |
| Health Motivation | | | | |
| 1. I am willing to spend some extra money for my health. | Agree | Somewhat agree | Somewhat disagree | Disagree |
| 1. I do everything I can to stay healthy. | Agree | Somewhat agree | Somewhat disagree | Disagree |
| 1. We should spend some extra time for health. | Agree | Somewhat agree | Somewhat disagree | Disagree |
| 1. I want to put health first in my living. | Agree | Somewhat agree | Somewhat disagree | Disagree |
| Health Value | | | | |
|  | 0 | 1 | 2 | 3 |
| 1. Work and income are more important than health. | Agree | Somewhat agree | Somewhat disagree | Disagree |
| 1. I worry about my health only when I get sick. | Agree | Somewhat agree | Somewhat disagree | Disagree |
| 1. Hobbies and leisure activities are more important than health. | Agree | Somewhat agree | Somewhat disagree | Disagree |
| 1. Rather than prevent illness, it is just to cure when I get sick. | Agree | Somewhat agree | Somewhat disagree | Disagree |

The total score of the Health Interest Scale (HIS) is 0–36.

The HIS questions were developed and validated in Japanese and the study was conducted in Japanese and this translated version was provided to show the contents in English by the authors.

Additional File 2. The characteristics of Health Interest Scale (HIS) categories

|  | Total | The categories of health interest by HIS  N=29,377 | | | | |
| --- | --- | --- | --- | --- | --- | --- |
|  |  | No (HIS:0-16) | Low  (HIS:17-20) | Middle  (21-22) | Middle-high  (23-26) | High  (27-36) |
|  | N=29,377 | N=6,195 | N=7,362 | N=4,070 | N=6,689 | N=5,061 |
|  | N (%) | N (%) | N (%) | N (%) | N (%) | N (%) |
| Age, year (Mean, SD) | 47.9(17.9) | 40.9(14.8) | 44.2(16.8) | 48.4(17.4) | 52.2(18.1) | 56.0(18.3) |
| 39 or less years | 10457(36) | 2959(48) | 2959(48) | 3120(42) | 1374(34) | 1865(28) |
| 40－59 years | 9773(33) | 2482(40) | 2482(40) | 2638(36) | 1386(34) | 1998(30) |
| 60 or over years | 9147(31) | 754(12) | 754(12) | 1604(22) | 1310(32) | 2826(42) |
| Sex |  |  |  |  |  |  |
| Men | 14389(49) | 3439(56) | 3792(52) | 1925(47) | 3020(45) | 2213(44) |
| Women | 14988(51) | 2756(44) | 3570(48) | 2145(53) | 3669(55) | 2848(56) |
| Education |  |  |  |  |  |  |
| Low | 8858(30) | 2103(34) | 2205(30) | 1177(29) | 1938(29) | 1435(28) |
| Middle | 5977(20) | 1233(20) | 1472(20) | 890(22) | 1362(20) | 1020(20) |
| High | 14542(50) | 2859(46) | 3685(50) | 2003(49) | 3389(51) | 2606(51) |
| Marital status |  |  |  |  |  |  |
| Marriage | 16829(57) | 2787(45) | 4012(55) | 2448(60) | 4321(65) | 3261(64) |
| No marriage | 9875(34) | 2909(47) | 2764(38) | 1271(31) | 1734(26) | 1197(24) |
| Divorced/Widowed | 2673(9) | 499(8) | 586(8) | 351(9) | 634(9) | 603(12) |
| Living alone | 6557(22) | 1695(27) | 1687(23) | 848(21) | 1282(19) | 1045(21) |
| Job |  |  |  |  |  |  |
| Executive/management | 2973(10) | 646(10) | 840(11) | 430(11) | 632(9) | 425(8) |
| Permanent employee | 8688(30) | 2312(37) | 2529(34) | 1189(29) | 1653(25) | 1005(20) |
| Self-employee | 1658(6) | 340(5) | 390(5) | 219(5) | 377(6) | 332(7) |
| No-regular employee | 5096(17) | 1128(18) | 1292(18) | 741(18) | 1160(17) | 775(15) |
| Students | 2057(7) | 549(9) | 588(8) | 284(7) | 386(6) | 250(5) |
| Retirement | 919(3) | 72(1) | 148(2) | 143(4) | 270(4) | 286(6) |
| Houseworker | 4474(15) | 519(8) | 898(12) | 654(16) | 1283(19) | 1120(22) |
| Unemployed | 3512(12) | 629(10) | 677(9) | 410(10) | 928(14) | 868(17) |
| Income |  |  |  |  |  |  |
| Under 2 million yen | 4601(16) | 1101(18) | 1131(15) | 588(14) | 977(15) | 804(16) |
| 2-4 million yen | 10239(35) | 2068(33) | 2499(34) | 1452(36) | 2441(36) | 1779(35) |
| 4-6 million yen | 4856(17) | 981(16) | 1259(17) | 682(17) | 1134(17) | 800(16) |
| 6-10 million yen | 2974(10) | 516(8) | 715(10) | 416(10) | 698(10) | 629(12) |
| 10 million or more | 628(2) | 108(2) | 143(2) | 65(2) | 157(2) | 155(3) |
| Don't know/Don't want to answer | 6079(21) | 1421(23) | 1615(22) | 867(21) | 1282(19) | 894(18) |

Additional File 3. The Alcohol Use Disorders Identification Test (AUDIT) and each score of answers

|  | 0 | 1 | 2 | 3 | 4 |
| --- | --- | --- | --- | --- | --- |
| 1. How often do you have a drink containing alcohol? | Never | Monthly or less | 2 to 4 times a month | 2 to 3 times a week | 4 or more times a week |
| 1. How many drinks containing alcohol do you have on a typical day when you are drinking? | 1 or 2 | 3 or 4 | 5 or 6 | 7, 8, or 9 | 10 or more |
| 1. How often do you have six or more drinks on one occasion? | Never | Less than monthly | Monthly | Weekly | Daily or almost daily |
| 1. How often during the last year have you found that you were not able to stop drinking once you had started? | Never | Less than monthly | Monthly | Weekly | Daily or almost daily |
| 1. How often during the last year have you failed to do what was normally expected from you because of drinking? | Never | Less than monthly | Monthly | Weekly | Daily or almost daily |
| 1. How often during the last year have you needed a first drink in the morning to get yourself going after a heavy drinking session? | Never | Less than monthly | Monthly | Weekly | Daily or almost daily |
| 1. How often during the last year have you had a feeling of guilt or remorse after drinking? | Never | Less than monthly | Monthly | Weekly | Daily or almost daily |
| 1. How often during the last year have you been unable to remember what happened the night before because you had been drinking? | Never | Less than monthly | Monthly | Weekly | Daily or almost daily |
| 1. Have you or someone else been injured as a result of your drinking? | No |  | Yes, but not in the last year |  | Yes, during the last year |
| 1. Has a relative or friend or a doctor or another health worker been concerned about your drinking or suggested you cut it down? | No |  | Yes, but not in the last year |  | Yes, during the last year |

The total score of AUDIT is 0-40.

Additional File 4. Means and standard deviations of Health Interest Scale (HIS) according to drinking categories

|  | Non-drinker （0） | Low-risk （1-7） | Medium-risk (8-14) | High-risk (15-19) | Likely alcohol dependence (20-40) |  |
| --- | --- | --- | --- | --- | --- | --- |
|  | N=9,700 | N=15,325 | N=2,841 | N＝779 | N＝732 |  |
|  | Mean (SD) | Mean (SD) | Mean (SD) | Mean (SD) | Mean (SD) | P for trend* |
| Health interest score by each factor in HIS | 21.0(6.0) | 21.4(5.7) | 20.7(5.9) | 19.5(5.3) | 18.6(5.6) | <0.001 |
| Factor1: health consciousness | 7.1(3.0) | 7.4(2.8) | 7.2(2.8) | 6.7(2.6) | 6.2(2.9) | <0.001 |
| Factor2: health motivation | 6.6(2.7) | 6.9(2.4) | 6.6(2.5) | 6.3(2.4) | 6.0(2.8) | <0.001 |
| Factor3: health value | 7.3(2.4) | 7.1(2.3) | 6.9(2.3) | 6.5(2.3) | 6.3(2.5) | <0.001 |

*The test of a linear trend was conducted for the score of HIS and drinking categories.
